# Therapeutic potential of hydrogen-rich water in zebrafish model of Alzheimer’s disease: targeting oxidative stress, inflammation, and the gut-brain axis

**DOI:** 10.3389/fnagi.2024.1515092

**Published:** 2025-01-07

**Authors:** Jiaxuan He, Peiye Xu, Ting Xu, Haiyang Yu, Lei Wang, Rongbing Chen, Kun Zhang, Yueliang Yao, Yanyan Xie, Qinsi Yang, Wei Wu, Da Sun, Dejun Wu

**Affiliations:** ^1^Institute of Life Sciences and Biomedical Collaborative Innovation Center of Zhejiang Province, Wenzhou University, Wenzhou, China; ^2^Technical Institute of Physics and Chemistry, Chinese Academy of Sciences, Beijing, China; ^3^Department of Biomedical Engineering, City University of Hong Kong, Kowloon, Hong Kong SAR, China; ^4^Chongqing Municipality Clinical Research Center for Geriatric Diseases, Chongqing University Three Gorges Hospital, Chongqing, China; ^5^Fuzhou Innovation Center for AI Drug, Fuzhou Medical College of Nanchang University, Fuzhou, China; ^6^The Affiliated Kangning Hospital of Wenzhou Medical University, Wenzhou, China; ^7^Wenzhou Institute, University of Chinese Academy of Sciences, Wenzhou, China; ^8^Key Laboratory for Biorheological Science and Technology of Ministry of Education, State and Local Joint Engineering Laboratory for Vascular Implants, Bioengineering College, Chongqing University, Chongqing, China; ^9^Department of Geriatric Medicine, Quzhou People’s Hospital, Quzhou, China

**Keywords:** Alzheimer’s disease, zebrafish, hydrogen-rich water, behavior, antioxidant, reduced Aβ deposition, modulated liver sEH activity, alleviated neuroinflammation and oxidative stress

## Abstract

Alzheimer’s disease (AD) is a complex neurodegenerative disorder, with amyloid-beta (Aβ) aggregation playing a key role in its pathogenesis. Aβ-induced oxidative stress leads to neuronal damage, mitochondrial dysfunction, and apoptosis, making antioxidative strategies promising for AD treatment. This study investigates the effects of hydrogen-rich water (HRW) in a zebrafish AD model. Zebrafish were exposed to aluminum chloride to induce AD-like pathology and then treated with HRW using a nanobubble device. Behavioral assays, ELISA, Hematoxylin–eosin (H&E) staining, and reactive oxygen species (ROS) and neutrophil fluorescence labeling were employed to assess HRW’s impact. Additionally, 16S rRNA sequencing analyzed HRW’s effect on gut microbiota. HRW can significantly improve cognitive impairment and depression-like behavior in zebrafish AD model, reduce Aβ deposition (*p* < 0.0001), regulate liver Soluble epoxide hydrolase (sEH) levels (*p* < 0.05), reduce neuroinflammation, and reduce oxidative stress. Furthermore, HRW reduced the number of harmful bacteria linked to AD pathology by restoring the balance of microbiota in the gut. These findings suggest that HRW has potential as a therapeutic strategy for AD by targeting oxidative stress, inflammation, and gut-brain axis modulation.

## 1 Introduction

As global populations continue to age, neurodegenerative diseases, particularly Alzheimer’s disease (AD), have become significant public health concerns, with profound social and economic implications (Scheltens et al., 2021). In 2024 alone, approximately $360 billion will be spent on healthcare and associated services for AD patients (Alzheimer’s Association, 2024). As AD progresses, it imposes increasing burdens on families and communities, making it a major public health crisis (Benek et al., 2015).

AD is pathologically characterized by the accumulation of amyloid-beta (Aβ) plaques and neurofibrillary tangles composed of hyperphosphorylated tau protein (Rahman and Lendel, 2021). Aβ, a central player in AD pathogenesis, not only forms toxic aggregates but also contributes to oxidative stress, a key factor in the progression of neurodegenerative processes (Ono et al., 2023; Bai et al., 2022). Oxidative stress leads to mitochondrial dysfunction and the excessive production of reactive oxygen species (ROS), exacerbating the accumulation of Aβ and further intensifying the oxidative damage (Tamagno et al., 2012; Forman and Zhang, 2021), This creates a vicious cycle that drives the progression of AD. Additionally, neuroinflammation, marked by the upregulation of pro-inflammatory cytokines such as Interleukin-1 beta (IL-1β), Interleukin-6 beta (IL-6) and tumor necrosis factor (TNF-α), further amplifies neuronal damage. Astrocytes and microglia, the primary immune cells of the brain, are central to this chronic neuroinflammation response, which is a hallmark of AD (Leng and Edison, 2021).

Emerging research has also underscored the significant influence of gut-brain axis in AD pathology. Dysbiosis of gut microbiota is linked to increased oxidative stress and neuroinflammation, contributing to AD progression (Qu et al., 2024). Certain gut metabolites, including short-chain fatty acids, play protective roles by modulating ROS levels. However, dysregulated microbial metabolites, such as nitric oxide (NO), can disrupt the intestinal barrier and promote systemic inflammation, exacerbating AD-related neuroinflammation (Liu et al., 2021; Shandilya et al., 2022; Wang et al., 2020).

Currently approved drugs for the treatment of Alzheimer’s disease (AD) mainly include cholinesterase inhibitors (such as Donepezil, rivastigmine, galantamine) and NMDA receptor antagonists (such as Memantine) (Wu et al., 2023). The medications are designed to improve cognitive functions and daily activities, they do not modify the disease, and they often cause serious side effects such as cardiovascular problems (such as bradycardia), severe digestive symptoms, and even seizures (Grossberg, 2003; Zhang et al., 2023; Vossel et al., 2017). It is therefore imperative that new treatments with minimal side effects and positive effects on health are developed.

Current therapeutic strategies for AD, including antioxidant therapies (Kamaljeet Singh et al., 2024), have yielded inconsistent results and sometimes adverse effects. For instance, high doses of vitamin E have been associated with an increased risk of heart failure in elderly patients, raising concerns about the safety and efficacy of such interventions (Lloret et al., 2019; Pérez-Bermúdez et al., 2017). In light of these limitations, there is a growing interest in exploring alternative therapeutic approaches, such as the potential use of hydrogen (H_2_).

H_2_ has a very small molecular weight, is electrically neutral, colorless, tasteless, has some reducibility, and has excellent diffusion properties. It has the ability to pass through cell membranes, organelle membranes, and play its biological role (Ghaffari-Bohlouli et al., 2024). A number of studies have shown that interventions based on hydrogen, such as inhaling and drinking water rich in hydrogen, and using hydrogen carriers with a slow release, are beneficial to AD patients (He et al., 2023). Nevertheless, animal models are primarily based on mice, and no simultaneous exploration of the efficacy evaluation and mechanism elucidation of H_2_ on the main pathological features of AD (neuroinflammation, oxidative stress, disturbance of gut flora) is undertaken.

Zebrafish (*Danio rerio*), with their high genetic homology to humans and well-established use in neurodegenerative disease research, offer a valuable model for studying AD (Wang et al., 2023). Recent studies have shown that zebrafish can effectively mimic key aspects of AD pathology, making them a suitable alternative to traditional rodent models (Saleem and Kannan, 2018; Qu et al., 2023). In addition, the aquatic environment of zebrafish allows for efficient absorption of hydrogen through hydrogen-rich water, providing a novel avenue for exploring the therapeutic potential of H_2_ in neurodegenerative diseases. However, conventional methods of delivering hydrogen, such as direct injection or hydrogen gas infusion, are prone to gas loss and may cause stress in zebrafish, potentially leading to inconsistent experimental results (Kawamura et al., 2020; Demin et al., 2021).

**Figure 1:**
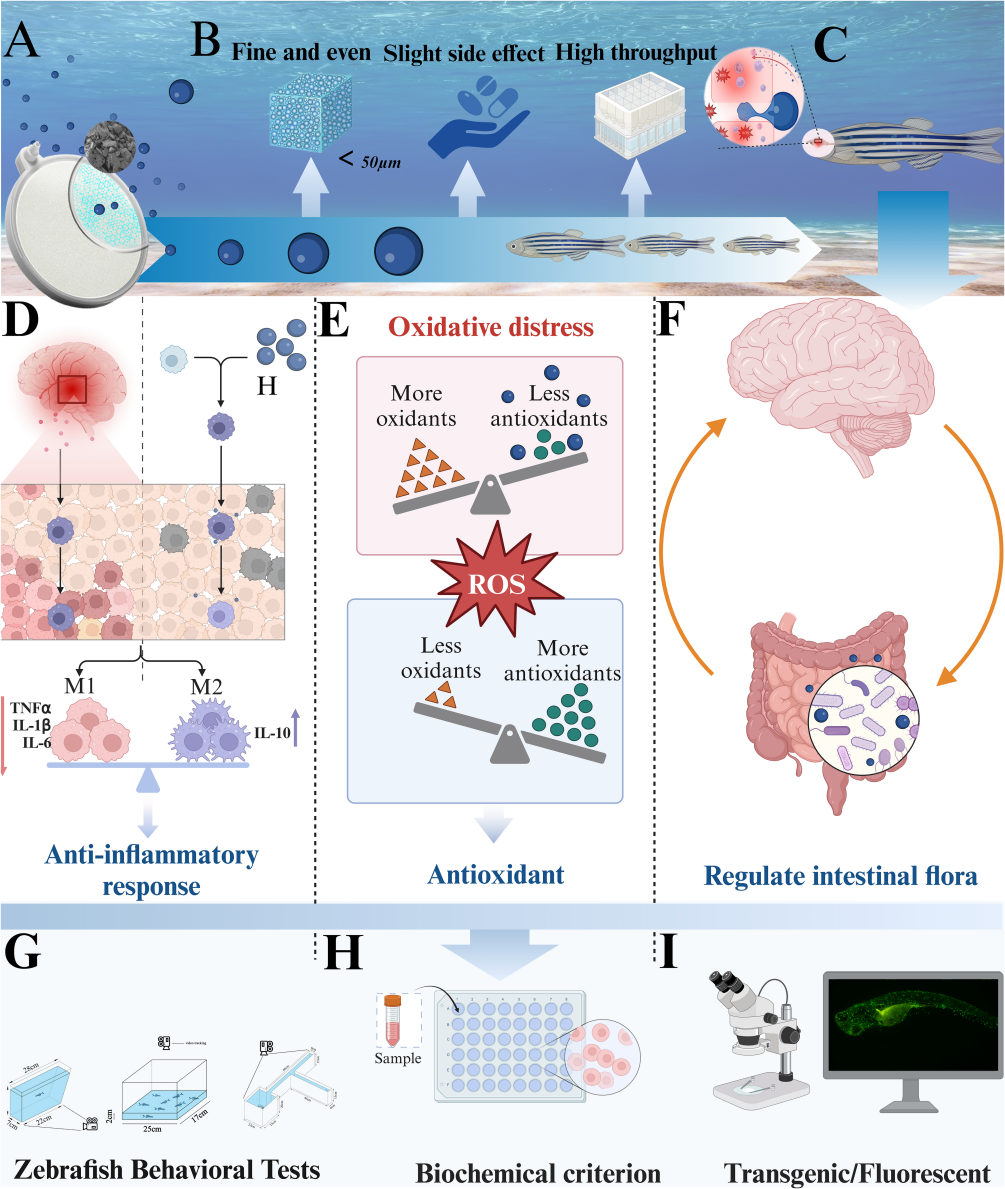
Experimental strategy and mechanism. **(A)** Nano-bubble disk. **(B)** Hydrogen emission from nano-bubbles. **(C)** Molecular hydrogen blood–brain barrier penetration. **(D)** The anti-inflammatory effect of hydrogen. **(E)** The antioxidant effect of hydrogen. **(F)** The regulation of intestinal flora by hydrogen. **(G)** Behavioral tests. **(H)** Biochemical index detection. **(I)** Fluorescence observation of zebrafish larvae.

To address these challenges, we developed a nanobubble device capable of delivering hydrogen evenly and stably into the zebrafish environment, minimizing stress and optimizing hydrogen absorption (Figure 1). Using this approach, our study aimed to evaluate the protective effects of hydrogen-rich water (HRW) on AD-like pathology in zebrafish, focusing on its ability to alleviate oxidative stress, reduce neuroinflammation, and modulate gut microbiota. By integrating these dimensions, we provide a comprehensive exploration of hydrogen’s therapeutic potential in the context of Alzheimer’s disease.

## 2 Materials and methods

### 2.1 Zebrafish maintenance and growth conditions

Wild-type zebrafish (Department AB, *Danio rerio*) and genetically modified zebrafish (*rj30Tg/+AB*) are maintained at the Institute of Life Sciences, School of Life and Environmental Sciences, Wenzhou University. Zebrafish maintained at a temperature of 28.5 ± 0.5°C and a light/dark period of 14 h/10 h (light intensity: 54–324 lux), dissolved oxygen ≥6.0 mg/L, pH 6.9–7.3, conductivity 500–800 μS/cm, salinity 0.25–0.75% in the circulatory system (Chen et al., 2023). After male and female fish mate one-on-one, fertilized eggs are collected and raised in incubators at 28°C. Feeding method and medium composition of juvenile fish: 50 embryos were cultured in approximately 120 mL E3 medium (5 mM NaCl, 0.17 mM KCl, 0.33 mM CaCl_2_, 0.33 mM MgSO_4_) with 120 μL/L 0.1% methylene blue added. The embryos were kept in sodium-calcium glass petri dishes with a diameter of 4.5 inches, and the dishes were kept at 28.5°C using an incubator, the dishes were checked daily, the media were changed, and unfertilized eggs and non-viable embryos were removed (Norton et al., 2019). The raised zebrafish were randomly divided into three groups (120 in each group, 7 months of age) with a sex ratio of 1:1 in each group: Control group (CG), AD group, HRW group. Control group were fed under normal conditions, AD and HRW groups were treated with aluminum chloride for 1 month, and HRW was soaked with aluminum chloride for 1 month and then treated with HRW for 7 days.

### 2.2 Construction and measurement of hydrogen-rich environment

**Figure 2:**
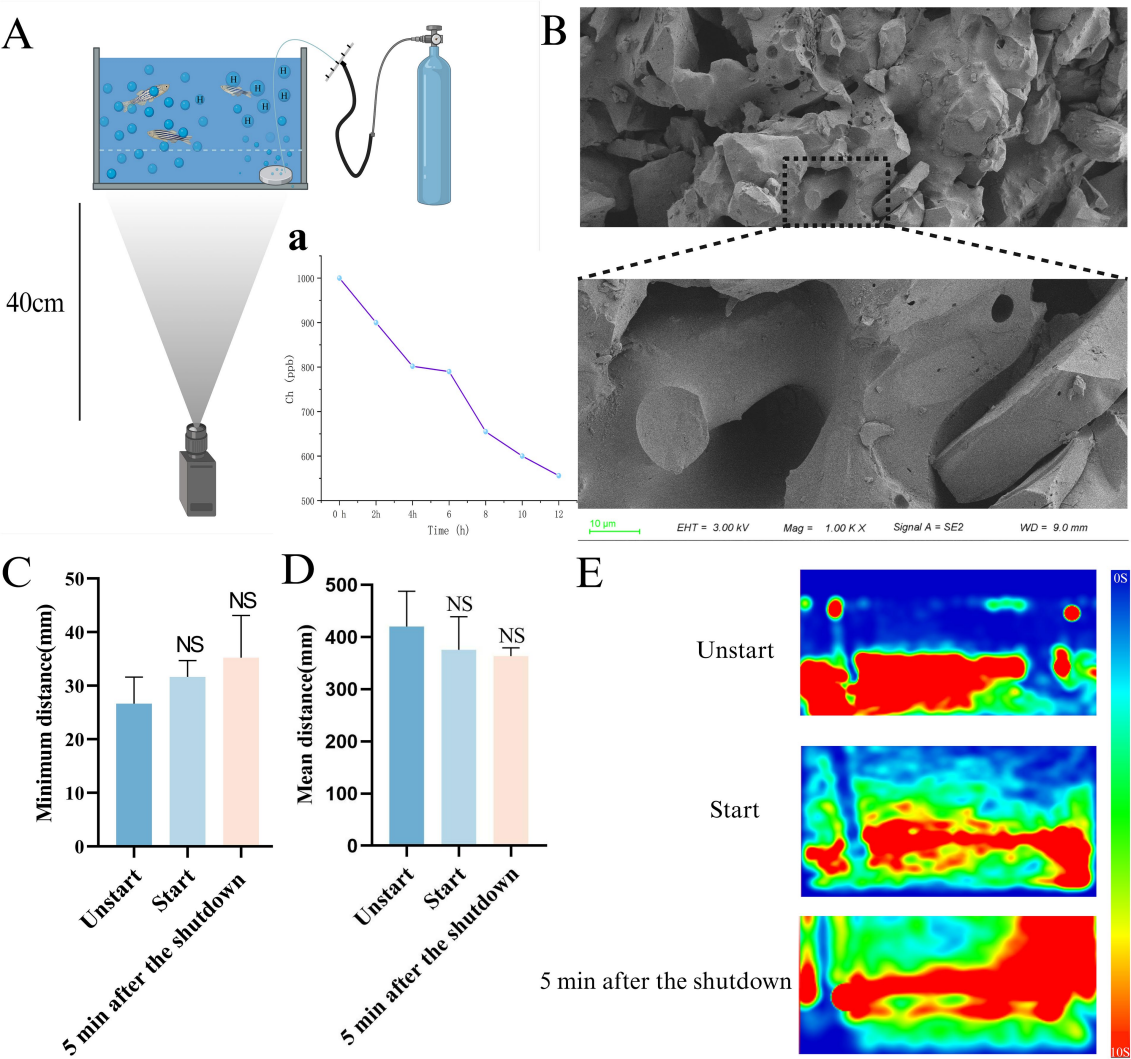
Device component schematic and safety verification. **(A)** Schematic diagram of the capture device. (a) The change in hydrogen concentration in the unit over a 12-h period. **(B)** SEM image of nano-bubble disk. **(C)** Minimum distance between individuals. **(D)** Mean distance. **(E)** The Trace heat maps of unstart group, start group, 5 min after the shutdown (*n* = 10 per group). The data are expressed as the mean ± SEM and were analyzed by one-way ANOVA followed by Tukey’s post-hoc test. Significance was defined as follows versus unstart group: NS - not significant.

The nano-bubble tray (Zhuoxing Technology Co., LTD., China) was placed in A zebrafish aquarium, and then the other end was connected to a voltage divider to enable it to better control the hydrogen flow rate. The terminal was connected to a high-purity hydrogen bottle for hydrogen supply, as shown in Figure 2A. Hydrogen was supplied at a constant speed of 5 MPa for 20 min, and a hydrogen-rich test pen was used for real-time monitoring (EpU Instrument, China) to make its hydrogen concentration reach 1,000 ppb, and the change of hydrogen concentration in the aquarium was measured at 0, 2, 4, 6, 8, 10, 12 h (Figure 2A).

### 2.3 Construction of zebrafish AD model

According to the laboratory’s previous experience in building AD zebrafish (Qu et al., 2023), the zebrafish was exposed to 500 μg/L aluminum chloride solution, the pH was adjusted to 5.8 with dilute hydrochloric acid, and one-third of the poison solution was changed every day for 30 days. Zebrafish are purchased from the China Zebrafish Resource Center and cared for, bred, fed, and challenged in strict compliance with the National Institutes of Health Guidelines for the Care and Use of Laboratory Animals.

### 2.4 Hydrogen-rich water intervention

According to Stokes Law, smaller diameter bubbles generally mean higher dissolution efficiency (Manica et al., 2016), Thanks to its microporous structure, nanobubble disks can produce fine bubbles with a diameter of less than 50 μm. After only 20 min of release, the concentration of HRW in the solution was able to reach a near-saturation concentration (1,000 ppb), allowing follow-up studies to be conducted.

General environment (control group and AD group): Culture environment temperature 28°C, PH:7.35, no additional hydrogen. HRW environmental parameters (HRW group): the hydrogen concentration in the aquarium is 1,000 ppb, and other parameters are consistent with the general environment. The HRW group was placed in a hydrogen-rich environment for 10 h and a normal environment for 14 h, and the other two groups were placed in a normal environment all day, and the intervention lasted for 7 days in all three groups. The research also verified the relaxation characteristics of the device. Through behavioral capture analysis, the camera was located 40 cm away from the device, and the device-on group (Start) and the device-not-on group (Unstart) were filmed for 5 min each, respectively. The individual distance and average swimming distance of zebrafish would increase under stress (Demin et al., 2021; Moreira et al., 2024; Kirsten et al., 2021).

Therefore, comparing the minimum swimming distance and mean distance between individuals in the start group and the unstart group became a means to detect whether zebrafish in the open group were in a state of stress. In addition, considering the possible influence of stress after the completion of hydrogen injection, we also conducted behavioral photographing of zebrafish 5 min after the shutdown the completion of hydrogen injection, using the same method as Shoaling test.

### 2.5 Behavioral testing

#### 2.5.1 T-maze

**Figure 3:**
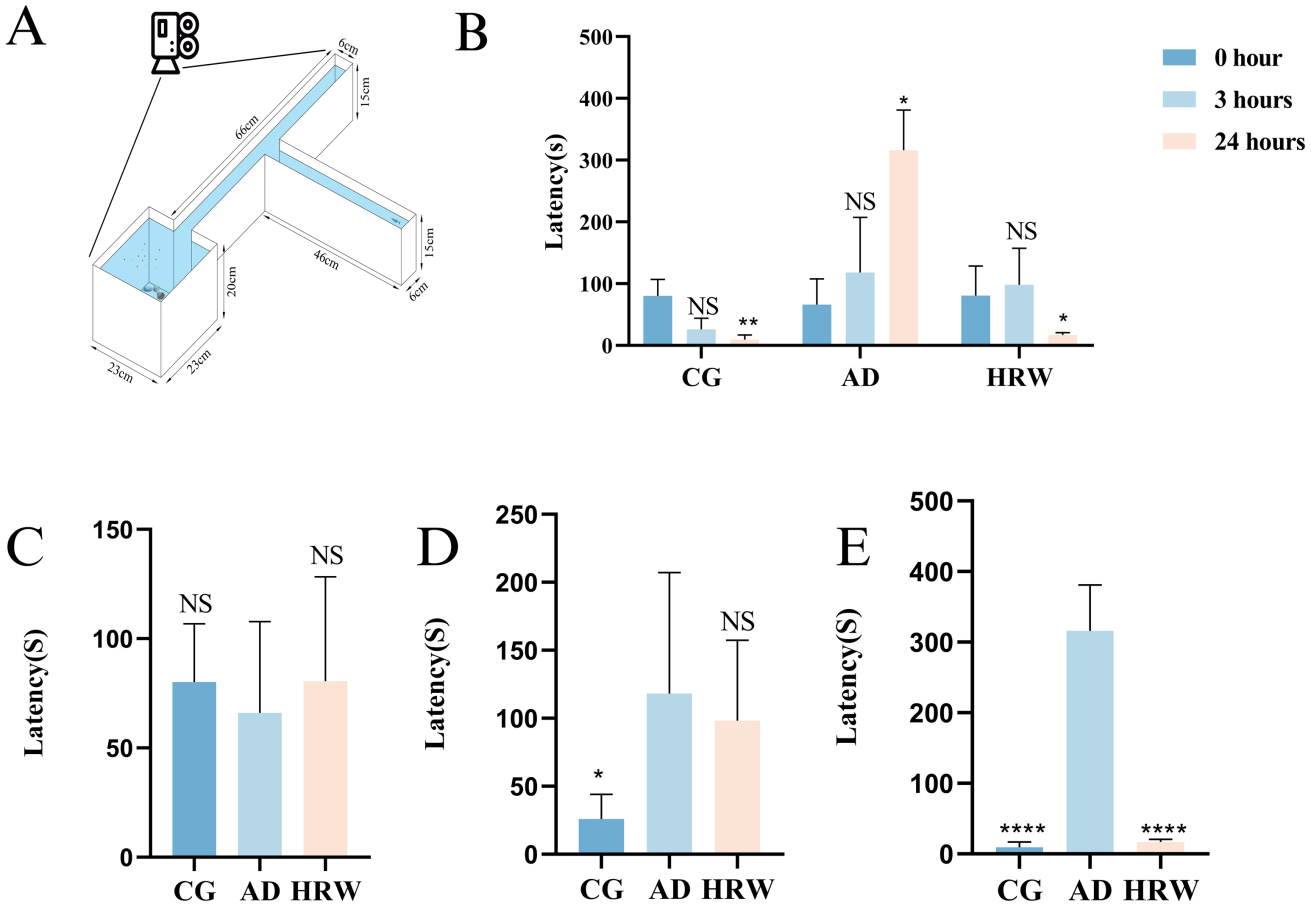
**(A)** Diagram of T-maze device. **(B)** Time trend diagram of zebrafish finding deep water area in different groups. **(C)** Time it takes the zebrafish to find deep water at 0 h. **(D)** Time it takes the zebrafish to find deep water at 3 h. **(E)** Time it takes the zebrafish to find deep water at 24 h (*n* = 5 per group). The data are expressed as the mean ± SEM and were analyzed by one-way ANOVA followed by Tukey’s post-hoc test. Significance was defined as follows versus AD group: *p < 0.05, **p < 0.01, ****p < 0.0001. NS, not significant.

Since the occurrence of AD is often accompanied by cognitive dysfunction (Zhang et al., 2024), we used a T-maze to test zebrafish behavior. The T-maze consists of a long, straight, horizontal arm, divided into two shorter arms connected at a 90° Angle, one of which has a slightly deep water area at the end of the short arm, containing shells, stones and feed (Figure 3A). The memory of zebrafish was evaluated according to the description of Ma et al., and rationalized improvements were made (Ma et al., 2024). The deep water region is black, while the other region is white. The time it took for zebrafish to enter deep water for the first time and stay there for at least 20 s was recorded. The memory tests were performed at 0, 3, and 24 h.

#### 2.5.2 Noval tank test (NTT)

**Figure 4:**
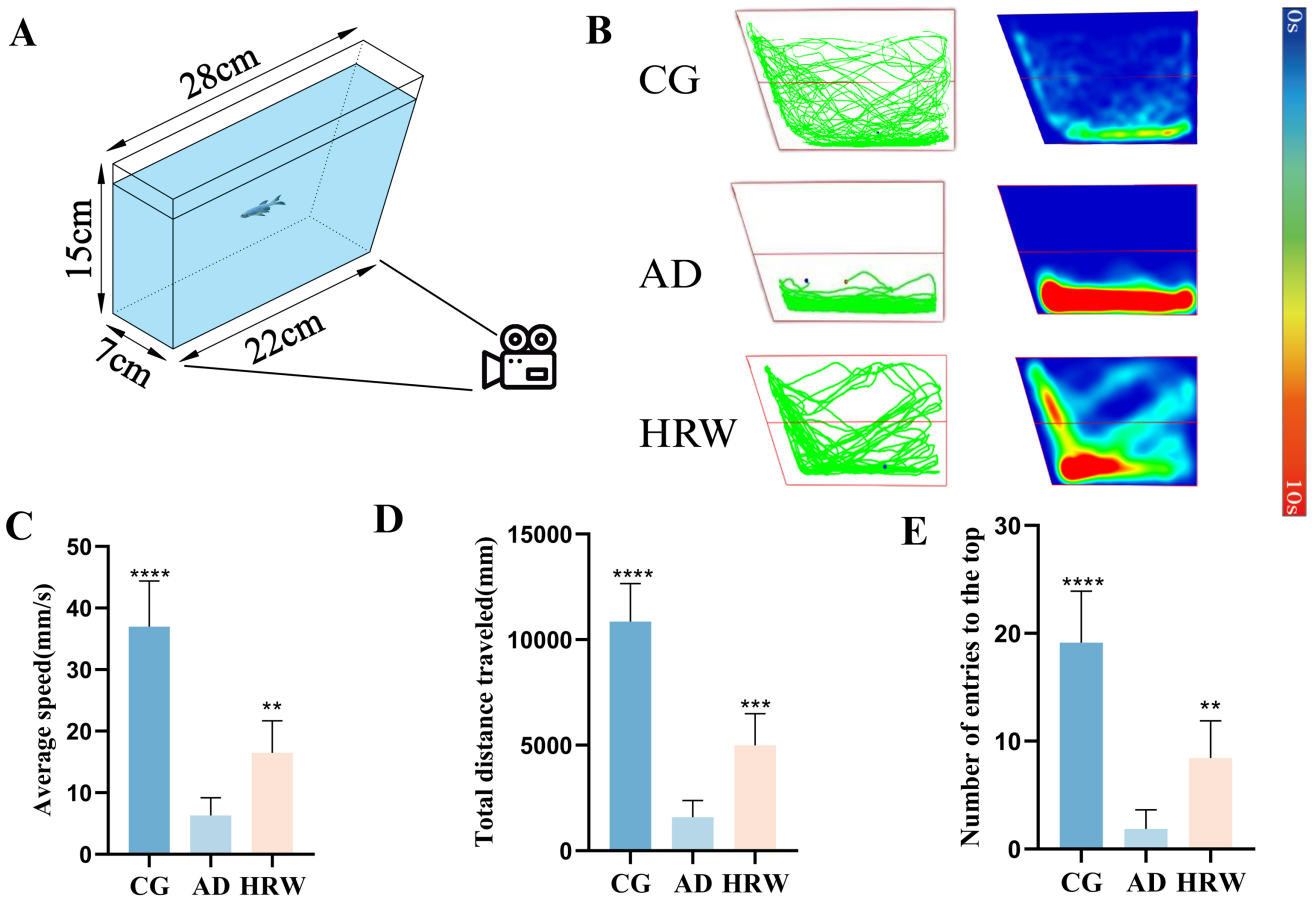
**(A)** Schematic diagram of NTT device. **(B)** Track chart and heat map of each group during capture time. **(C)** The average speed of each group. **(D)** The total distance traveled. **(E)** The number of entries the top. NTT (*n* = 7 per group). The data are expressed as the mean ± SEM and were analyzed by one-way ANOVA followed by Tukey’s post-hoc test. Significance was defined as follows versus AD group: *p < 0.05, **p < 0.01, ****p < 0.0001. NS, not significant.

AD individuals often exhibit depressed-like behavior, which has been found in experiments with mice and zebrafish (Chen et al., 2021; Botto et al., 2022; Fang et al., 2021). NTT provides an assessment of the level of depression in zebrafish, and Depressed individuals generally show more residence time in the lower end area, slower swimming speed and shorter swimming distance (Brinza et al., 2023; Boiangiu et al., 2023). A tank (28 × 7 × 15 cm) was filled with 15 cm of water. Vertically, the water surface is evenly divided into upper and lower parts (Figure 4A). The fish was placed in the test tank for 2 min to acclimatize, then filmed and recorded its movements for 5 min. According to the improvement of the specific behavior of the experiment, the number of zebrafish entering the top, the average speed (mm/s) and the total distance traveled (mm) were analyzed and determined to evaluate the depressed-like behavior of zebrafish.

#### 2.5.3 Shoaling test

**Figure 5:**
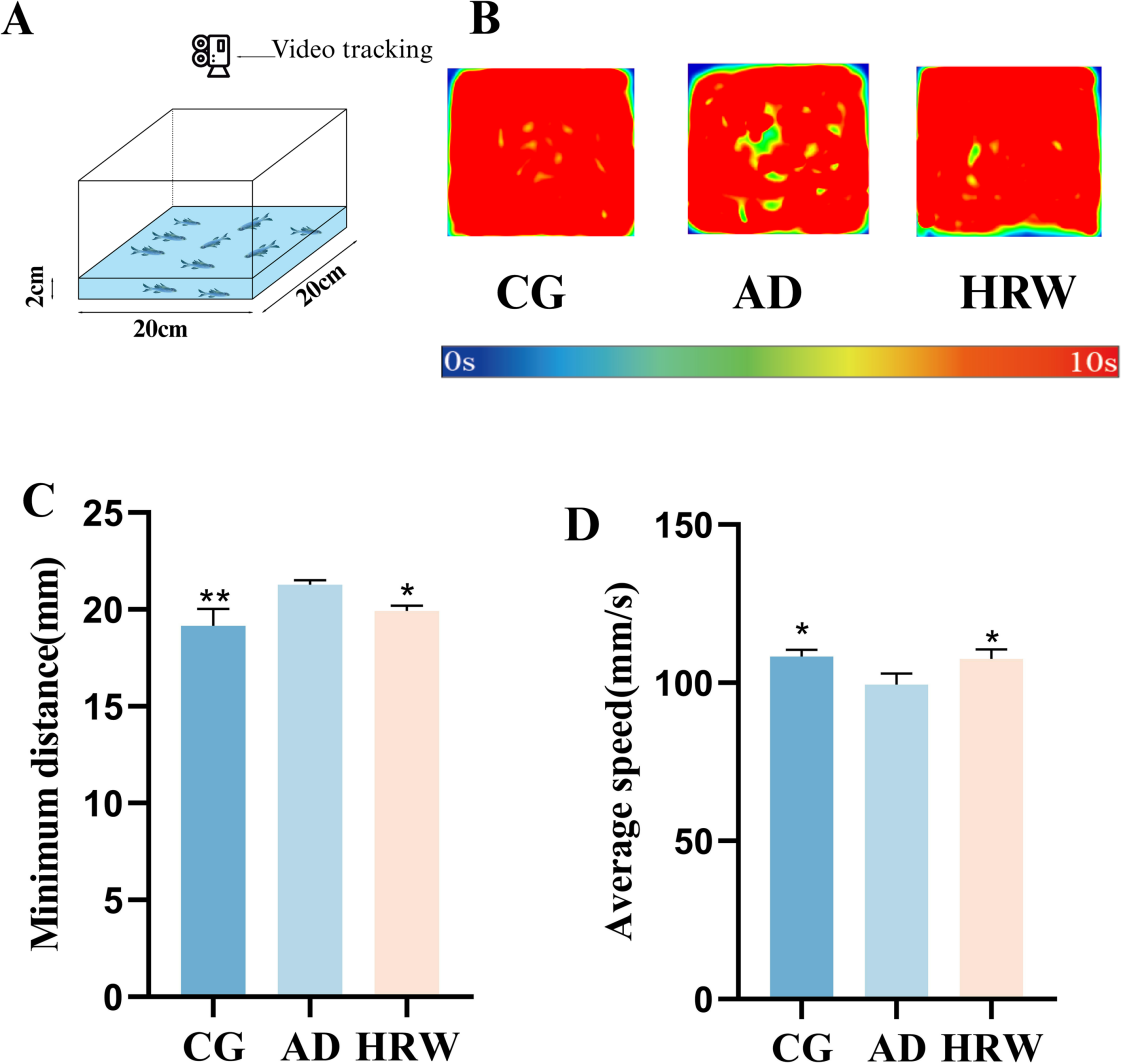
**(A)** Schematic diagram of the shoal installation. **(B)** Track heat map of each group during capture time. **(C)** Minimum distance between individuals. **(D)** Average speed (*n* = 10 per group). The data are expressed as the mean ± SEM and were analyzed by one-way ANOVA followed by Tukey’s post-hoc test. Significance was defined as follows versus AD group *p < 0.05; **p < 0.01.

Shoal preference is defined as an individual choosing to be close to animals of the same species. Zebrafish are a highly social species, and shoal preference can be observed by their response to social stimuli. Zebrafish in depressive condition generally produce social avoidance behavior, which is characterized by an increase in distance between individuals and a decrease in average speed (Ogi et al., 2020; Wang et al., 2022; Johnson et al., 2023). According to WANG’s design (Wang et al., 2022), The number of zebrafish with *n* = 10 was placed in the length, width and height (20 cm × 20 cm × 10 cm), and the water level was 2 cm (Figure 5A), and the trajectory of their activity in the device for 10 min was recorded. The following motor parameters were measured: the average speed, and the minimum distance between individuals.

In order to test whether the device would stimulate zebrafish, the analytical experiment was carried out in the same way as the Shoaling test, Compare the mean distance and the minimum distance between start groups with 5 min after the shutdown and unstart groups to test the safety of their devices.

### 2.6 Brain biochemical analysis and hematoxylin–eosin (H&E) staining

In addition to amyloid accumulation in the brain of zebrafish with AD, inflammation and oxidative stress also occur (Zhang et al., 2024). In this experiment, the brains and livers of control, AD and HRW groups (*n* = 12 for each group) were dissected from the killed animals. The fresh tissues were homogenized in 0.9%PBS (1:9 [wt/wt]) and centrifuged at 5,000×*g* at 4°C. The supernatant was collected for biochemical determination. Aβ1-42 in brain and Soluble epoxide hydrolase (sEH) in liver were detected by enzyme-linked immunosorbent assay (ELISA) kit. Biochemical detection kit (Solarbio) was used to determine the oxidative stress index of brain samples: catalase (CAT), glutathione (GSH), Malondialdehyde (MDA) superoxide dismutase (SOD). And the levels of interleukin 6 (IL-6), interleukin 10 (IL-10), interleukin 1β (IL-1β), and TNF-α in brain samples (ZIKER, Nanjing, China).

Brain tissue was dissected from zebrafish, fixed in formalin, embedded in paraffin blocks, and used a microtome (RM-2125 RT; Leica, Germany). Sections on microscope slides are first dewaxed in xylene, rehydrated in gradient alcohol, and stained with hematoxylin and eosin (H&E) dye (Sigma-Aldrich). After rinsing with running water, the tissue sections were soaked in alcohol and xylene successively to dehydrate. The stained sections were then installed in xylophenol medium (Muto pure chemical, Tokyo, Japan) and xylene, and the brain inflammation and edema and neuronal status were observed under an optical microscope (NIKON ECLIPSE E100, Nikon, Shanghai, China).

### 2.7 Neutrophil and juvenile ROS staining

24 hpf fertilized eggs were divided into a control group, an AD group, and an HRW group, *n* = 3 in each group. Control group: Standard juvenile medium was cultured for 7 days. AD group: Embryo medium with a concentration of 50 μg/L AL^3+^ was treated, soaked for 4 days, and then transferred to normal medium for 3 days. HRW group: The medium containing 50 μg/L AL^3+^ was soaked for 4 days, and then transferred to the medium configured with 1,000 ppb HRW for 3 days of intervention. We then updated the culture medium daily for a duration of 3 days. On the 5th, 6th, and 7th days, we examined and photographed each batch of zebrafish larvae using a fluorescent microscope. We then analyzed the fluorescence intensity of the brain using ImageJ software. The fluorescence intensity of neutrophils in the brain of juvenile zebrafish was measured.

The fertilized eggs incubated on the same day were divided into Control, AD, and HRW groups (*n* = 3 for each group), and the intervention method was the same as above. On the 7th day, ROS enrichment of AB strain zebrafish was detected with 2′,7′-dichlorodihydrofluorescein diacetate (DCFH-DA) as probe. Using embryo fluid, DCFH-DA was diluted to a workable solution of 10 μmol/L. After 30 min of incubation at 28°C in complete darkness in a 6-well plate containing 10 μmol/L DCFH-DA, the embryos were rinsed three times with embryo solution to detect ROS. We captured the images using an inverted fluorescent microscope (Olympus IX73, Japan).

### 2.8 16S rRNA sequencing and analysis

In order to evaluate the differences in the intestinal flora between the treatment group and the AD group and the control group, three samples were collected from each group, each sample had 15 intestines, and the genome of the bacterial colony was extracted from the intestinal contents for amplification, and the recovered products were amplified using fluorescence quantitative polymerase chain reaction. The V3–V4 hypervariable regions of 16 S rDNA were amplified (forward: ACTCCTACGGGAGGCAGCA, reverse: GGACTACCAGGGTATCTAATCCTGTT), and the amplified products were then sequenced by the Illumina platform. DADA2 method mainly included primer removal, quality filtering, denoising, splicing, and clustering. Greengenes database was selected for species annotation. Sequencing service was provided by Shanghai Personal Biotechnology Co., Ltd., China. The data were analyzed by using the free online platform Personalbio GenesCloud.

### 2.9 Statistical analysis

Standard Errors of the Mean (SEM) of all data are expressed as group mean ± SEM. The data were analyzed using one-way analysis of variance (ANOVA). All statistical analyses were performed using GraphPad for Windows version 8 (GraphPad Prism Software Inc., San Diego, CA, USA). A *p* value <0.05 was considered statistically significant. All figures show significance level is as follows: *p < 0.05, **p < 0.01, ***p < 0.001, ****p < 0.0001.

## 3 Results

### 3.1 The dispersal device does not cause oxidative stress behavior of zebrafish

Due to the hydrogen launcher’s use of microporous nanobubble disks during launch (Figures 2A,B) during the emission period, the thermal map generated by the trajectory route, the mean distance and the minimum distance between individuals were captured and analyzed compared with that of the non-opened device group. There was no significant difference between the two groups (start groups, 5 min after the shutdown) and that of the unstart group in Figures 2C,E, Movies S1:1–3.

### 3.2 HRW improved cognitive dysfunction and depression-like behavior in AD-like zebrafish

The results of T-MAZE showed that HRW improved cognitive function relative to AD group, at 0 h, 3 h, 24 h and the time to find the deep water zone was shortened. AD group showed an extended time to reach the deep water zone (Figures 3B–D, Movies S2:1–9), and HRW improved cognitive function in AD group. In addition, both Control and HRW groups showed shorter arrival times in 24 h compared with AD (Figure 3E, Movies S2:1–9).

NTT results showed that, compared with the control group, AD group showed a slowdown in average speed, a decrease in total swimming distance, and a decrease in the number of times entering the upper region (Figures 4B–E, Movies S3:1–3), showing depression-like behavior, while HRW group showed an improvement in depression-like behavior.

Shoaling test results show that AD group shows social avoidance behavior compared with control group. According to the heat map, it can be intuitively seen that the social distance gap in AD group center is larger. According to the captured video analysis: Compared with control group, the minimum distance between individuals in AD group increased and the average speed decreased, while the HRW treatment group was improved (Figures 5B–D, Movies S4:1–3).

### 3.3 HRW can attenuate brain inflammation and promote brain nerve recovery in AD group

**Figure 6:**
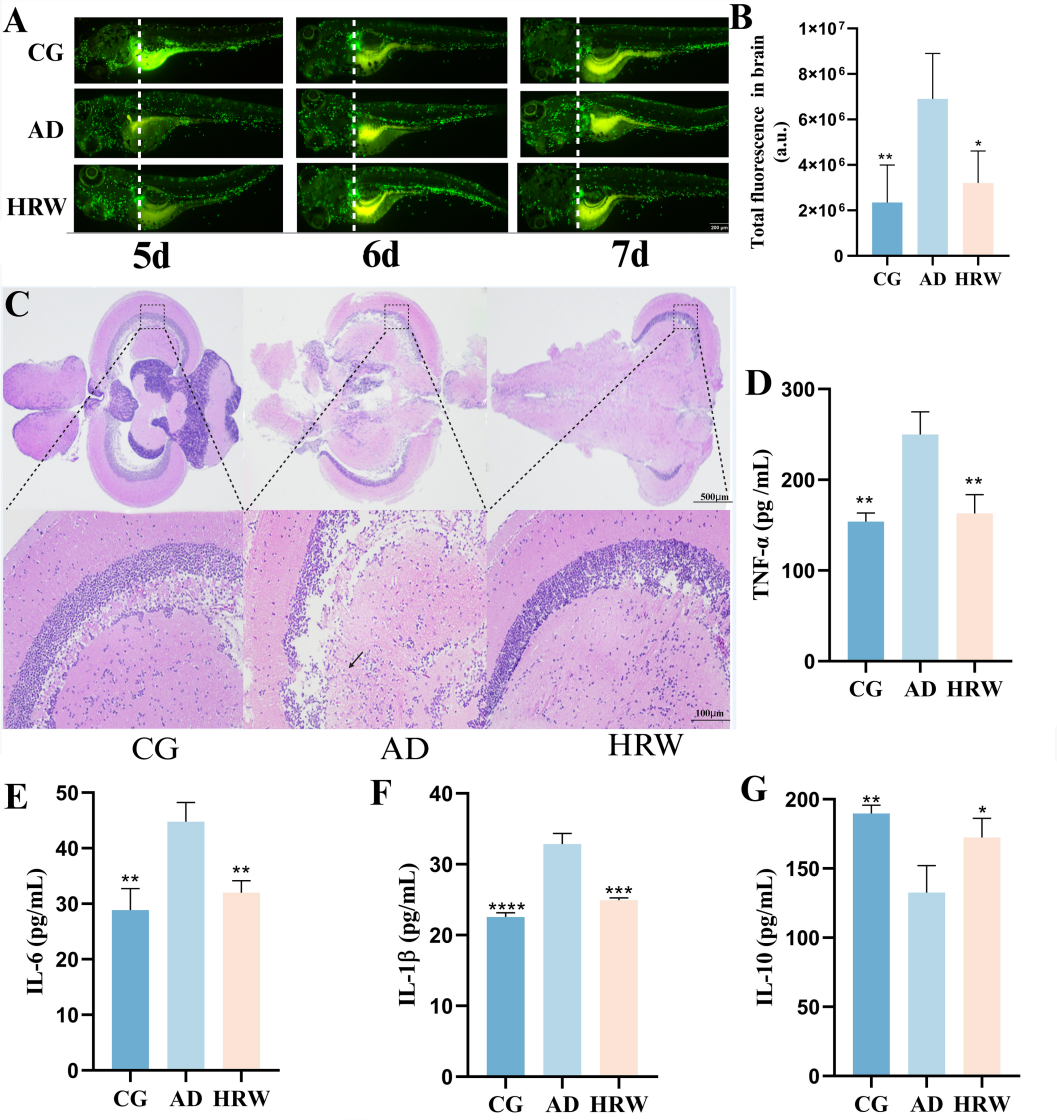
According to different zebrafish groups, the brain neutral particle fluorescence intensity, whole brain pathological section and whole brain tissue inflammatory factor levels. **(A)** Fluorescent aggregation of neutrophils in the brain of zebrafish (*n* = 3 per group). **(B)** Total fluorescence intensity. **(C)** Brain H&E staining, **(D)** TNF-α. **(E)** IL-6. **(F)** IL-1β. **(G)** IL-10 (*n* = 12 per group). The data are expressed as the mean ± SEM and were analyzed by one-way ANOVA followed by Tukey’s post-hoc test. Significance was defined as follows versus AD group *p < 0.05; **p < 0.01; ***p < 0.001; and ****p < 0.0001.

The aggregation of neutrophils is often accompanied by the occurrence of inflammation (Gour et al., 2024), which is consistent with the stronger fluorescence results observed in the brains of the AD group, while the fluorescence in the brains of zebrafish treated with HRW was reduced (*p* < 0.05) in Figures 6A,B. H&E staining results showed neuronal degeneration, a small amount of inflammatory infiltration, and cytoplasmic vacuolation in the brains of the AD group, while the HRW treatment group improved these pathological conditions in Figure 6C. Biochemical indicators showed that pro-inflammatory factors IL-6 (*p* < 0.01), IL-1β (*p* < 0.001), and TNF-α (*p* < 0.01), decreased, while the anti-inflammatory factor IL-10 increased (*p* < 0.05) in the brains of zebrafish treated with HRW in Figures 6D–G, indicating that HRW improved the inflammatory microenvironment in the brains under AD conditions.

### 3.4 After HRW treatment, zebrafish decreased amyloid precipitation in brain and sEH in liver

**Figure 7:**
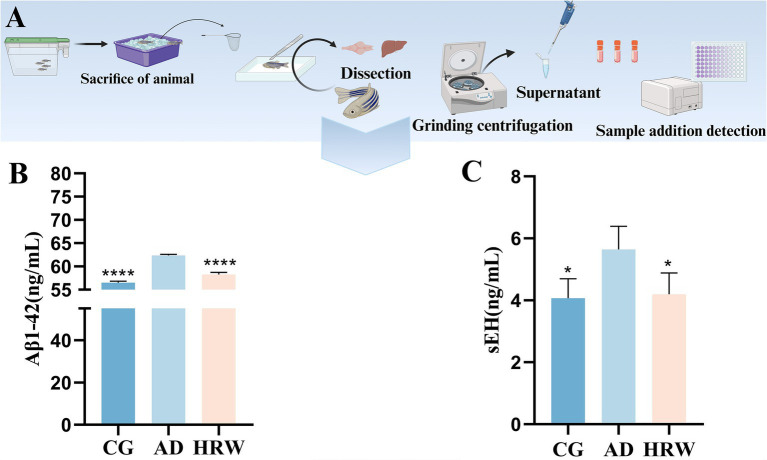
Detection of brain AD factor and liver sEHs in Zebrafish. **(A)** Inspection operation flow. **(B)** Aβ1-42. **(C)** sEH (*n* = 12 per group). The data are expressed as the mean ± SEM and were analyzed by one-way ANOVA followed by Tukey’s post-hoc test. Significance was defined as follows versus AD group: *p < 0.05, ****p < 0.0001.

Amyloid plaques are considered to be classic indicators of the occurrence of AD (Scheltens et al., 2021). Through the detection of biochemical indicators (Figure 7A)., we found more amyloid precipitation in the AD group. After HRW treatment, plaques in the brain of the treatment group were reduced (*p* < 0.0001) (Figure 7B). Amyloid plaques in the brain often coincide with high levels of sEH (*p* < 0.05) in the liver, and HRW treatment reduces their levels in the liver (Figure 7C).

### 3.5 HRW treatment modulated oxidative stress in zebrafish brain

**Figure 8:**
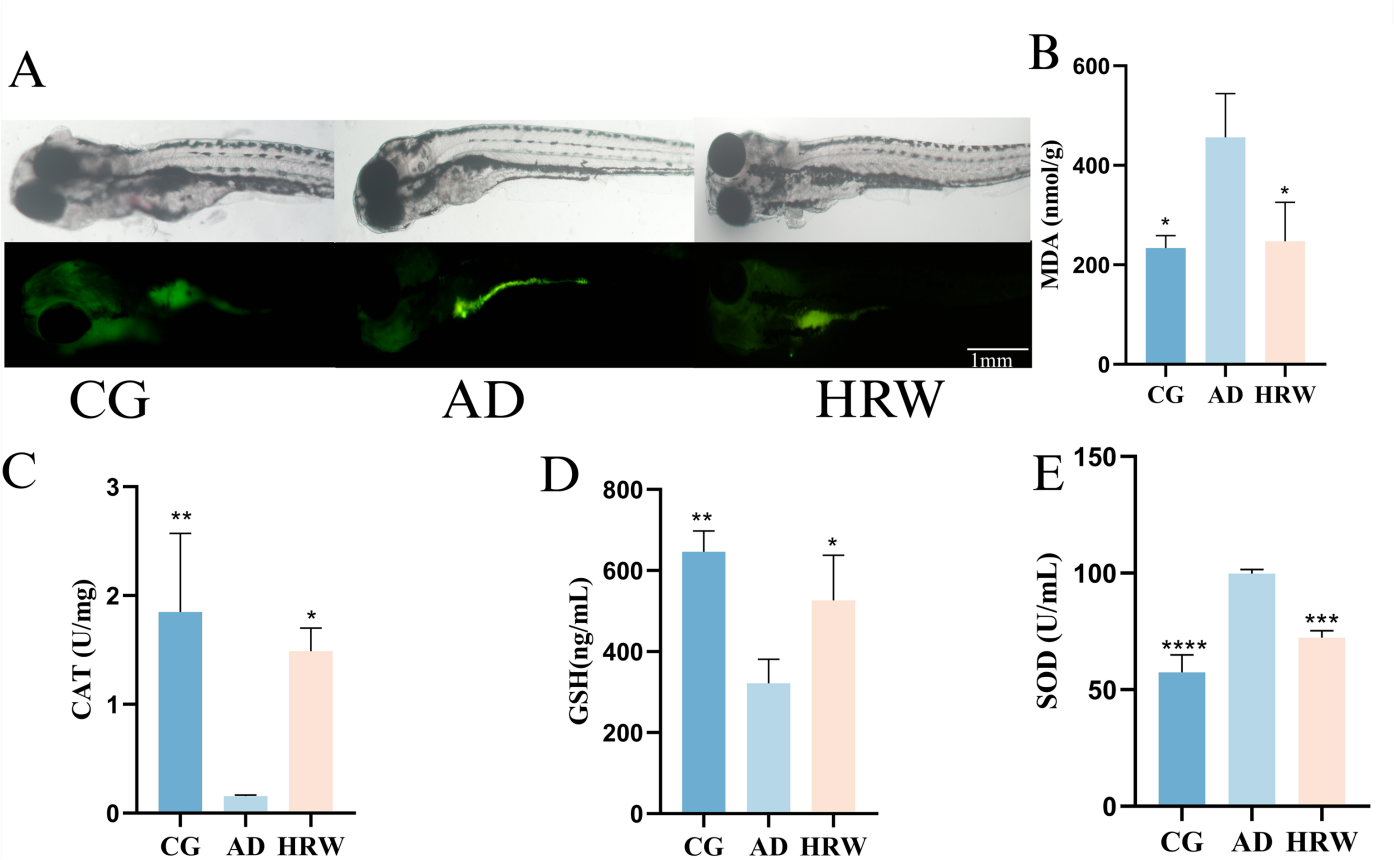
The whole brain oxidative stress factor and whole body ROS fluorescence intensity were analyzed according to different groups of zebrafish. **(A)** ROS juvenile fish dye fluorescence. **(B)** MDA. **(C)** CAT. **(D)** GSH. **(E)** SOD (*n* = 12 per group). The data are expressed as the mean ± SEM and were analyzed by one-way ANOVA followed by Tukey’s post-hoc test. Significance was defined as follows versus AD group *p < 0.05; **p < 0.01; ***p < 0.001; and ****p < 0.0001.

Oxidative stress, characterized by an imbalance between the production of free radicals and the body’s ability to combat their harmful effects, has been identified as an important factor in the pathogenesis of AD (Forman and Zhang, 2021). Oxidative damage was observed in the abdomen of the AD group and fluorescence in the abdomen of ROS stained juvenile fish was stronger than that in the control group, while fluorescence intensity was reduced in the HRW treated group (Figure 8A). HRW treatment also decreased the level of MDA (*p* < 0.05) in the brain (Figure 8B) and increased the levels of CAT (*p* < 0.05) and GSH (*p* < 0.05) antioxidant enzymes Figures 8C,D. Interestingly, we found increased SOD production in the AD group. This is not consistent with previous results, which may be due to the activation of antioxidant pathway (Li et al., 2024) in the early stage of oxidative stress, which further promotes the production of antioxidant enzymes, while the release of SOD (*p* < 0.05) in HRW is increased compared with that in control group (Figure 8E), which may indicate that hydrogen can not only directly participate in the antioxidant process, but also contribute to the process. It can also activate the activity of endogenous antioxidant enzymes (Wu et al., 2019), thus producing anti-oxidation effects.

### 3.6 HRW corrected the intestinal flora disturbance in AD zebrafish

**Figure 9:**
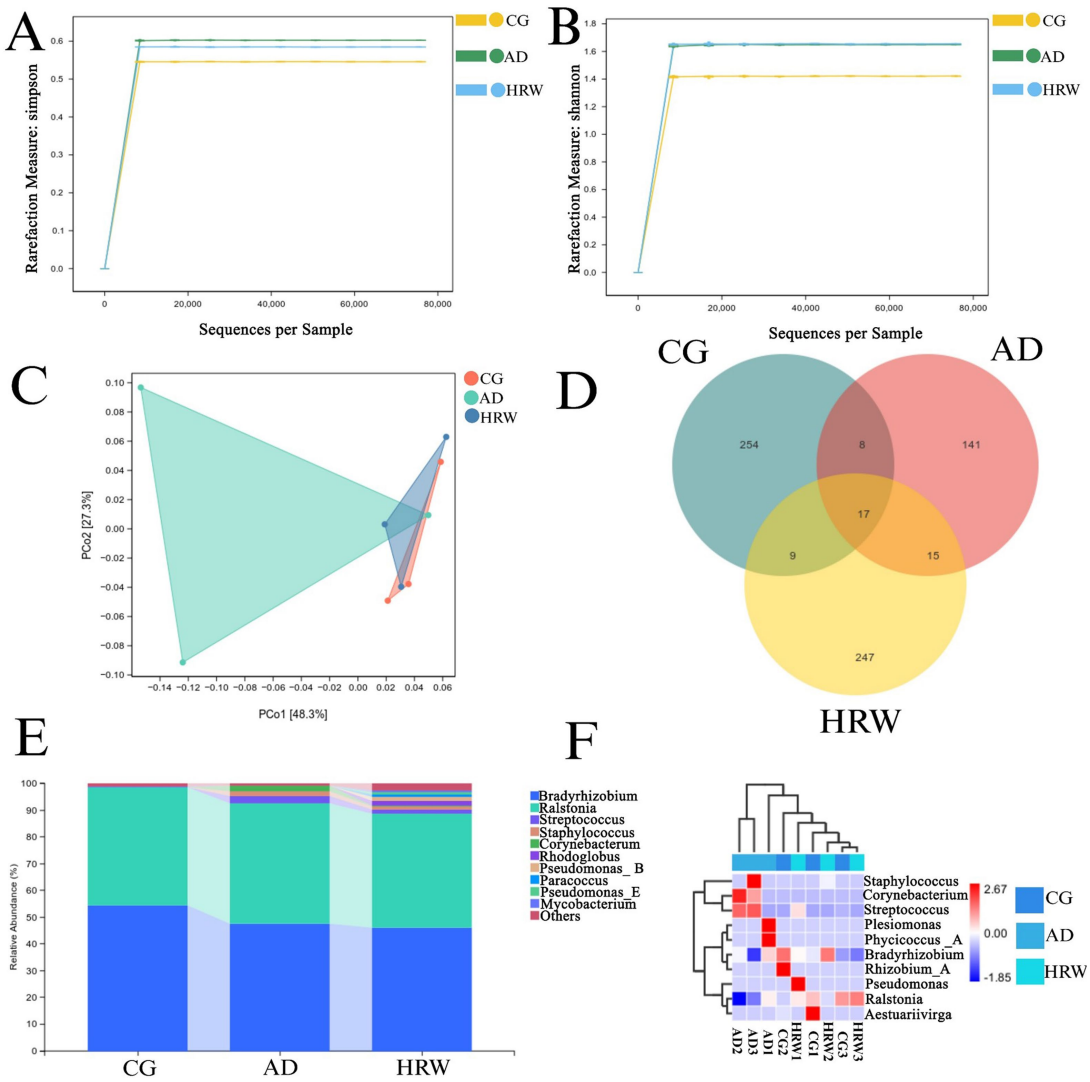
Effects of HRW on intestinal microorganisms of AD zebrafish. **(A)** Simpson index was used to characterize the diversity and uniformity of species distribution in zebrafish intestinal communities. **(B)** Shannon index was used to determine intestinal community diversity of zebrafish. **(C)** Comparison of intestinal flora β diversity between control group, AD group, and HRW group. **(D)** Venn diagram of species abundance. **(E)** Genus level intestinal microbial abundance. **(F)** Heat maps of the top 10 genera with average abundance were obtained by UPGMA clustering.

The intestinal flora diversity of AD zebrafish treated with HRW was observed by 16S rRNA sequencing. Each sequence is done as an OUT (classification operation unit), and each OUT corresponds to a typical sequence based on different similarity. The intestinal microbial composition of zebrafish in control group, AD group and HRW group was compared by cluster analysis of sequencing results. Analysis of α diversity (Shannon and Simpson indices) shows in Figures 9A,B, AD group had good community diversity after HRW treatment. The range of unique species in the three groups of samples was relatively diverse, indicating that the gut flora of zebrafish in each group varied greatly. A non-metric multidimensional analysis of beta diversity showed that the composition of microorganisms varied between groups. Distance matrix and PCoA analysis showed that the AD group was significantly different from the blank group (Figure 9C), This difference was improved after HRW treatment. Venn diagram showed that the diversity of bacteria in AD group was significantly lower than that in control and HRW group, this may be due to a decrease in gut probiotics in the AD group (Figure 9D). Heat maps were used to examine the average abundance of intestinal flora at the generic level in Figures 9E,F. According to the analysis, it has been found that *Ralstonia* (45.13%)*, Staphylococcus aureus* (*S. aureus*) (1.88%), *Streptococcus* (2.55%) and *Corynebacterium* (2.15%) in the AD group have increased in abundance. The bacterium Streptococcus and Staphylococcus have been shown to cause damage to the intestinal barrier, allowing endotoxins to enter the bloodstream, destroying the blood–brain barrier as well as causing neuroinflammation in order to damage nerve cells (Liu et al., 2022; Chelakkot et al., 2018; Brown, 2019), *S. aureus* also has the ability to produce Aβ protein (Bourgade et al., 2016; Lim et al., 2022), and the joint action of various harmful bacteria aggravates the deterioration of AD, After HRW treatment, the abundance of pathogenic bacteria was reduced (*Ralstonia* 42.63%*, S. aureus* 1.02%*, Streptococcus* 1.47%*, Corynebacterium* 0.51%).

## 4 Discussion

H_2_ has emerged as a promising therapeutic agent due to its ability to regulate oxidative stress, inflammation, and cellular metabolism, providing protective effects across various organs and systems, as demonstrated in both preclinical and clinical studies (Wu et al., 2018; Hu et al., 2022). Despite these advantages, hydrogen’s small molecules size and low solubility in water have limited its clinical application. To address this, we developed hydrogen nanobubbles using nanobubble disk emitters to enhance hydrogen solubility and stability in water. These nanobubbles maintain exceptional stability, allowing for continuous production of HRW at high concentrations, which supports prolonged therapeutic activity.

Given hydrogen’s well-documented antioxidant properties, including its ability to scavenge hydroxyl radicals and reduce oxidative damage, its therapeutic potential has been explored in conditions ranging from multi-organ ischemia/reperfusion injury to neurodegenerative diseases like AD, osteoarthritis, and respiratory diseases (Yuan et al., 2023). In this study, we specifically investigated the effects of HRW on AD pathology in a zebrafish model. AD is characterized by increased neuroinflammation, oxidative stress, and Aβ deposition, all of which contribute to neuronal dysfunction and cognitive impairment (Leng and Edison, 2021; Zhang et al., 2023; Lu et al., 2024).

Our biochemical analyses confirmed that HRW significantly decreased pro-inflammatory cytokine levels (TNF-α, IL-6, IL-1β) and increased IL-10 levels in zebrafish brains, suggesting that HRW exerts a strong anti-inflammatory effect. H&E staining revealed that HRW treatment alleviated brain inflammation and reduced neuronal degeneration. The observed reduction in neutrophil infiltration further corroborates these findings, demonstrating HRW’s ability to modulate inflammatory responses in the AD model.

Oxidative stress is a well-known driver of AD pathogenesis (Varesi et al., 2023), promoting Aβ accumulation and mitochondrial dysfunction. Our results are consistent with previous studies that highlight HRW’s antioxidative capacity. HRW treatment significantly reduced ROS levels and MDA concentration in the zebrafish brain, indicating its ability to mitigate oxidative damage (Ionescu-Tucker and Cotman, 2021). This was further supported by the observed increase in antioxidative enzymes such as CAT and GSH, which are essential in maintaining redox balance.

A critical finding of this study was the reduction of Aβ deposition in HRW-treated zebrafish, which suggests a potential link between oxidative stress modulation and Aβ dynamics. Given that oxidative stress exacerbates Aβ aggregation, the reduction in oxidative markers could explain the diminished Aβ deposition. Another important mechanism involves sEH, an enzyme that regulates the metabolism of neuroprotective epoxyeicosatrienoic acids. Inhibition of sEH has been shown to decrease Aβ production and deposition, improving cognitive outcomes in AD models (Wu et al., 2023; Sun et al., 2021), Our findings suggest that HRW may interact with sEH, stabilizing EET levels, thus mitigating oxidative stress and Aβ accumulation. This bidirectional regulation of sEH by HRW could be key to its neuroprotective effects, providing new insights into hydrogen’s role in AD therapy.

Recent studies have shown that the gut microbiota plays a significant role in the development and progression of neurodegenerative diseases, including AD (Thu Thuy Nguyen and Endres, 2022; Zhang et al., 2023). Dysbiosis of gut microbiota contributes to systemic inflammation, oxidative stress, and altered immune responses, all of which exacerbate AD pathology. Our study found that HRW treatment significantly altered the gut microbial composition in AD zebrafish, restoring β-diversity and reducing the abundance of pathogenic bacteria such as *Streptococcus*, *Staphylococcus*, and *Corynebacterium*. These bacterial strains are known to impair the integrity of the intestinal barrier, allowing endotoxins to enter the bloodstream and promote neuroinflammation.

In addition to its role in reducing intestinal inflammation, HRW may provide hydrogen to the intestine, enhancing the growth of beneficial bacteria and fostering a more balanced microbiota. This modulation of gut flora could contribute to the systemic anti-inflammatory effects observed in HRW-treated zebrafish. The gut-brain axis is increasingly recognized as a key pathway in neurodegeneration, and our findings suggest that HRW can modulate this axis by regulating gut microbiota composition, thereby reducing systemic and central inflammation.

The mechanisms by which HRW exerts its therapeutic effects in AD involve complex interactions between oxidative stress regulation, inflammatory modulation, and gut microbiota rebalancing. Our findings provide a comprehensive understanding of these interactions, positioning HRW as a promising therapeutic agent for neurodegenerative diseases like AD. However, several questions remain regarding the long-term effects of HRW and the precise molecular pathways involved.

Future studies should focus on multi-omics approaches, including transcriptomics, proteomics, and metabolomics, to fully elucidate the therapeutic mechanisms of hydrogen in AD. The effective concentration of HRW found in the Zebrafish study can provide a reference for dose optimization in mammals for long-term studies in mammalian models to observe the lasting effects of HRW on the pathological changes of Alzheimer’s disease. Additionally, translating these findings to clinical trials will be crucial in determining the broader applicability of HRW as a treatment for AD. The interactions between hydrogen, sEH regulation, Aβ dynamics, and gut microbiota offer exciting avenues for future research, with the potential to uncover novel therapeutic strategies for AD and other neurodegenerative diseases.

**Figure 10:**
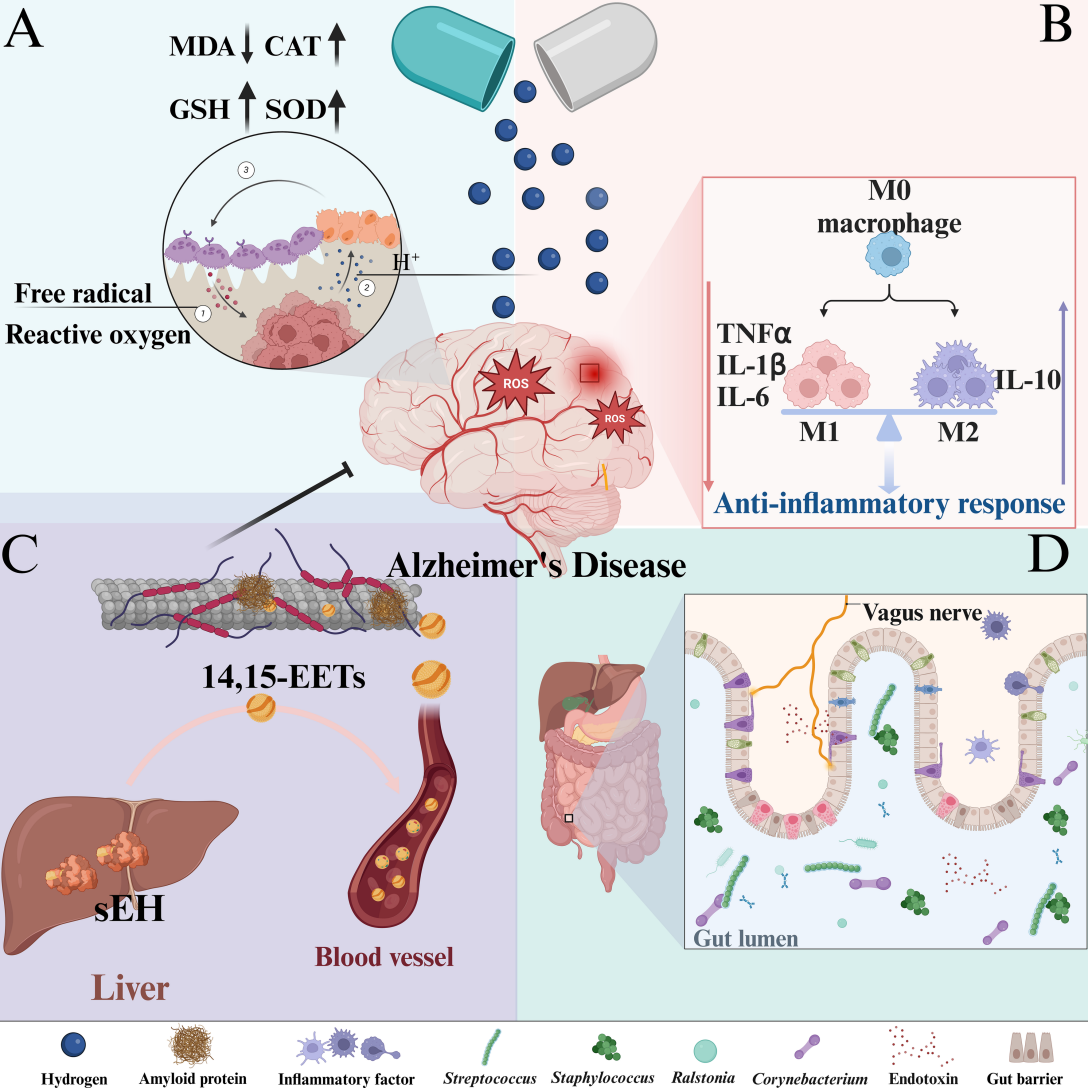
The mechanism of hydrogen-based therapy for AD: **(A)** Antioxidant effect. **(B)** Anti-inflammatory effect. **(C)** Regulation of hepatic sEH level. **(D)** Regulation of intestinal flora homeostasis.

The mechanism of hydrogen in this paper is shown in Figure 10.

## 5 Conclusion

This study highlights the therapeutic potential of HRW in mitigating AD pathology in zebrafish. HRW treatment significantly reduced Aβ deposition in the brain and regulated liver sEH, improving oxidative stress, neuroinflammation, and neuronal degeneration. Furthermore, HRW corrected gut microbiota dysbiosis, reducing harmful bacteria and promoting a healthier intestinal environment.

Our findings underscore HRW as a promising, multi-targeted approach for AD, with the potential to address key disease mechanisms, including oxidative stress, inflammation, and gut-brain interactions. These results offer a strong foundation for future research on hydrogen-based therapies in neurodegenerative diseases.

## Data Availability

Publicly available datasets were analyzed in this study. This data can be found at: movies is available at figshare, accession numbers: https://figshare.com/s/f258fc5394a9e151665f.
